# Development and validation of the Chinese version of the conditional reasoning test for implicit aggression in college students

**DOI:** 10.3389/fpsyg.2025.1460499

**Published:** 2025-04-25

**Authors:** Kequn Chu, Fengshu Zhu

**Affiliations:** ^1^College of Physical Education, Yangzhou University, Yangzhou, China; ^2^College of Educational Science, Guangxi Science and Technology Normal University, Laibin, China

**Keywords:** implicit aggression conditional reasoning test for college students, implicit aggression, college students, validity, reliability

## Abstract

**Objective:**

This study aims to develop an implicit aggression conditional reasoning test suitable for college students and to test its reliability and validity.

**Methods:**

Based on an in-depth literature review and expert interviews, the research team identified the theoretical structure of college students’ implicit aggression and the initial items. Through methods such as exploratory factor analysis, confirmatory factor analysis, and reliability and validity testing, the final test scale was formulated and optimized.

**Results:**

The final implicit aggression conditional reasoning test for college students contained 18 items across six dimensions: hostility attribution bias, target degradation bias, potency bias, revenge bias, oppression bias, and social discounting bias. Confirmatory factor analysis indicated that the scale had good construct validity (χ^2^ = 333.82, df = 132, RMSEA = 0.08, NFI = 0.91, CFI = 0.92, GFI = 0.90, PGFI = 0.66); the total internal consistency coefficient of the scale was 0.90, and the test-retest reliability was 0.87. The internal consistency coefficients of the subscales ranged from 0.87 to 0.92, with test-retest reliabilities between 0.84 and 0.90. Additionally, the test demonstrated good criterion validity.

**Conclusion:**

The development of the implicit aggression conditional reasoning test for college students meets the initial theoretical predictions, and its reliability and validity indicators satisfy the requirements of psychometrics, providing an effective tool for assessing and researching implicit aggression among college students.

## Introduction

Aggressive behavior not only impacts the physical and psychological development of college students but also has profound implications for their future social adaptability (Xu et al., 2023; Caibin et al., 2023). Such behavior can lead to deteriorated interpersonal relationships and declining academic performance among college students, significantly affecting their psychological wellbeing (Ye et al., 2022; Yu et al., 2024; Thomas, 2019). If not promptly controlled and intervened, aggressive behavior can pose severe threats to campus safety and social stability (Didden et al., 2016; Weltens et al., 2021). Therefore, the measurement of aggressive behavior in college students holds significant theoretical and practical importance.

In many social psychology studies, aggression is typically defined as “behavior intended to harm another person who does not wish to be harmed” (Krahé, 2020; Baron and Richardson, 1994). This classic definition focuses on aggression at the behavioral level, such as physical assault or verbal violence (Anderson and Bushman, 2002). However, this study adopts a different concept, namely “implicit aggression.” Implicit aggression refers to unconscious aggressive tendencies within an individual, such as hostile emotions and aggressive intentions, which usually do not manifest in overt external behaviors. Unlike traditional measurement tools for explicit aggressive behavior, the conditional reasoning test developed in this study aims to reveal an individual’s implicit aggression tendencies through the reasoning process.

Commonly used tools for measuring aggressive behavior include the Buss-Durkee Hostility Inventory (BDHI), the Buss-Perry Aggression Questionnaire (AQ), and the Reactive-Proactive Aggression Questionnaire (RPQ). The BDHI is primarily used to measure an individual’s hostility and aggressive behavior, encompassing multiple dimensions such as anger, hostility, and jealousy, aiding researchers in comprehensively understanding an individual’s propensity for aggressive behavior (Buss and Durkee, 1957; Vassar and Hale, 2009). The Buss-Perry Aggression Questionnaire (AQ) is the most widely used tool for assessing aggression, including dimensions of physical aggression, verbal aggression, anger, and hostility, providing a comprehensive evaluation of an individual’s aggression (Buss and Perry, 1992). The Reactive-Proactive Aggression Questionnaire (RPQ) distinguishes between reactive and proactive aggression, with the former referring to aggression triggered by stimuli or provocation, and the latter referring to aggression initiated proactively to achieve a certain goal (Raine et al., 2006). These self-report scales have been validated in numerous studies (Zeferino et al., 2024; Chu and Zhu, 2022; Puhalla and McCloskey, 2020). However, self-report scales are highly subjective, and individuals often cannot truthfully reflect their aggression due to social desirability bias (James and LeBreton, 2010). Research also indicates that self-report scales are effective in measuring explicit aggression but less so for implicit aggression (James et al., 2022). Explicit aggression refers to observable aggressive actions such as physical conflicts or verbal abuse (Yu et al., 2024), while implicit aggression involves unobservable aggressive tendencies such as hostile emotions or aggressive intentions, encompassing attitudes and emotions in the unconscious (James and LeBreton, 2010). Therefore, the measurement of implicit aggression is relatively complex.

Research indicates that individuals often exhibit reasoning biases that justify their aggressive actions, making them appear logical and reasonable, a phenomenon known as “justification mechanisms” (Barksdale, 2007; Galić, 2016). According to the justification mechanism theory, James categorized individual reasoning biases into six types: hostile attribution bias, goal derogation bias, efficacy bias, retaliation bias, oppression bias, and social discounting bias. These six justification mechanisms represent the reasoning processes that occur prior to an individual’s aggressive actions, often unconsciously (James et al., 2005). Based on these six mechanisms, James et al. (2005) developed the conditional reasoning test for aggression (CRT-A), which includes 22 inductive reasoning items, each presenting a scenario where participants must reason and respond based on the given context. The CRT-A, by designing logical reasoning tests, induces participants to reveal their implicit motives and attitudes during the reasoning process (James et al., 2005; James and LeBreton, 2010). Unlike traditional self-report scales, the conditional reasoning test bypasses individuals’ self-defense mechanisms and avoids social desirability effects, assessing participants’ behavioral responses in specific situations. Studies have shown that the CRT-A can reveal implicit aggression in individuals within a professional environment (Bowler et al., 2013). To test the cross-cultural validity of the CRT-A, researchers translated it into multiple languages and conducted cross-cultural revisions. Studies have shown that the CRT-A has good reliability and validity when applied in Croatia, the Netherlands, Saudi Arabia, and South Korea (Lee, 2017; Galić et al., 2014; LeBreton et al., 2022; Bowler et al., 2013). In a study conducted in Croatia, Galić et al. (2014) reported that the CRT-A demonstrated high internal consistency, with Cronbach’s alpha reaching 0.85, and acceptable construct validity confirmed through confirmatory factor analysis (CFA). Similarly, LeBreton et al. (2022) examined CRT-A in the Netherlands, reporting CFA model fit indices of CFI = 0.93 and RMSEA = 0.06, which fall within the recommended thresholds for structural validity. In South Korea, Lee (2017) observed significant predictive validity, as CRT-A scores were positively correlated with workplace aggression (*r* = 0.45, *p* < 0.01) and counterproductive work behavior. These findings indicate that the conditional reasoning test is an effective tool for measuring individuals’ implicit aggression and has cross-cultural applicability, helping us comprehensively understand and evaluate individuals’ aggression. However, existing conditional reasoning tests focus on implicit aggression in the workplace, with all scenario descriptions pertaining to workplace contexts. There is a lack of conditional reasoning tests specifically designed for measuring implicit aggression in college students. Currently, there is also no CRT-A developed specifically for Chinese populations or Chinese college students. The existing CRT-A versions are based entirely on Western cultural contexts, and their scenario descriptions and reasoning mechanisms may not directly apply to the cultural environment and experiences of Chinese college students.

Developing or adapting the CRT-A for Chinese college students is of both theoretical and practical significance. From a theoretical perspective, college students are at a critical stage of socialization and personality development. Their cognitive biases and implicit aggression not only influence their behavioral choices but can also have a profound impact on peer relationships and the campus environment. Revising the CRT-A to suit the Chinese context can provide a more comprehensive understanding of the characteristics of implicit aggression and cognitive biases in Chinese college students, offering valuable insights for cross-cultural research on this topic. From a practical perspective, an adapted version of the CRT-A can be used as an early screening tool for implicit aggression, supporting the design of psychological health education programs and intervention strategies. Ultimately, this can promote the psychological wellbeing of college students and contribute to harmonious campus environments.

Building on the theory of defense mechanisms and the format of the CRT-A, this study aimed to develop a conditional reasoning test specifically designed to measure implicit aggression in Chinese college students and to evaluate its reliability and validity.

## Methodology

### Participants

Sample 1: A total of 500 college students were randomly selected from Jiangsu, Guangxi, and Sichuan provinces. After removing 55 questionnaires with incorrect, missing, or dishonest responses, 445 valid questionnaires remained, yielding a validity rate of 89.00%. The average age of the participants was 20.45 ± 2.32 years, including 210 males (average age 20.37 ± 2.41 years) and 235 females (average age 20.52 ± 2.24 years). This sample was used for exploratory factor analysis. Participants were randomly selected from a variety of universities across the three provinces, with a focus on ensuring a diverse representation of students from different academic backgrounds. The questionnaire was administered electronically using the Wenjuanxing online platform, allowing participants to complete the survey via their mobile devices. The sample represented a broad range of academic disciplines, including engineering, social sciences, business, and humanities, ensuring a well-rounded participant base.

Sample 2: A total of 900 college students were randomly selected from Beijing, Jiangsu, Guangxi, Shandong, and Sichuan provinces. After removing 40 questionnaires with incorrect, missing, or dishonest responses, 860 valid questionnaires remained, yielding a validity rate of 95.56%. The average age of the participants was 21.32 ± 1.98 years, including 400 males (average age 21.45 ± 2.05 years) and 460 females (average age 21.20 ± 1.92 years). This sample was used for confirmatory factor analysis and reliability and validity testing. The participants were randomly selected from universities in both metropolitan and rural areas to ensure a diverse geographical sample. Like Sample 1, the survey was conducted electronically via the Wenjuanxing platform, which allowed for efficient data collection on participants’ mobile devices. The participants came from various academic fields, including engineering, business, social sciences, and arts.

Sample 3: A convenience sample of 200 participants (95 males and 105 females) was selected from Sample 2 for retest reliability testing. The average age of the participants was 20.50 ± 2.36 years, with males averaging 20.48 ± 2.42 years and females averaging 20.52 ± 2.31 years. This subset of participants, drawn from the same universities as Sample 2, was chosen for its availability and willingness to participate in the follow-up testing. The retest was also administered electronically, ensuring consistency in the data collection process across all samples.

### Scale development

#### Scale dimensions and item compilation

The justification mechanism theory posits that individuals employ a series of psychological strategies to shield themselves from internal and external pressures and conflicts (Barksdale, 2007; Galić, 2016). According to this theory, James identified six primary justification mechanisms: hostile attribution bias, goal derogation bias, efficacy bias, retaliation bias, oppression bias, and social discounting bias (James et al., 2005). Specifically, hostile attribution bias involves interpreting others’ motives toward oneself as hostile or aggressive; goal derogation bias refers to individuals belittling the importance of a goal to reduce frustration and stress when they fail to achieve it, thereby maintaining self-esteem and self-worth; efficacy bias involves overestimating one’s abilities or influence in certain situations to protect against feelings of inadequacy, which can help maintain confidence in the face of challenges but may also lead to overconfidence and poor decision-making; retaliation bias is the tendency to believe that retaliating against unfair actions by others is justified and necessary, serving to uphold self-esteem and a sense of justice; oppression bias involves individuals perceiving themselves as victims of stronger external forces, helping to explain and cope with feelings of powerlessness and failure by attributing responsibility to external entities; social discounting bias involves underestimating or ignoring the contributions or value of others in social interactions, protecting against feelings of inferiority and stress induced by social comparisons.

Building on the justification mechanism theory, the conditional reasoning test for aggression (CRT-A) in a professional context sets the six justification mechanisms as six dimensions, compiling 22 conditional reasoning items. We emulated this approach by developing inductive reasoning items based on the six justification mechanisms specifically for college students, ultimately forming the initial version of the conditional reasoning test for implicit aggression in college students.

Expert Interviews: To ensure the accuracy and relevance of the items in the conditional reasoning test for implicit aggression in college students, we conducted expert interviews with a panel consisting of two psychology professors, two doctoral candidates, and five master’s students in psychology. These interviews were held in May 2023. During the interviews, the experts reviewed the initial items of the test, providing feedback on their clarity, appropriateness, and alignment with the theoretical framework of implicit aggression. The data collected from these interviews were analyzed qualitatively, and the feedback was used to revise and refine the questionnaire. The experts’ insights were integral to the final version of the scale, ensuring it effectively measured implicit aggression in college students.

#### Initial revision of the scale

After completing the initial version of the conditional reasoning test for implicit aggression in college students, we assembled a panel consisting of two psychology professors, two doctoral candidates, and five master’s candidates in psychology to review and evaluate the scale items. These expert interviews were conducted in May 2023, and their feedback was integral in refining the scale. The experts reviewed the clarity, relevance, and consistency of the items in relation to the theoretical framework of implicit aggression. Based on criteria including construct validity, clarity, conciseness, and comprehensiveness, we engaged in multiple discussions to eliminate items with redundant meanings or those inconsistent with their respective dimensions, and revised items with abstract or logically flawed expressions. The feedback from the experts was analyzed and used to revise the test items, ensuring they reflected implicit aggression accurately. After thorough deliberation, we finalized a version of the test comprising 18 items across six dimensions, with three items per dimension. Each item offers four options: one aggressive response, one prosocial response, and two illogical options. Participants receive a score of +1 for selecting the aggressive response, −1 for the prosocial response, and 0 for choosing an illogical option. Detailed examples of the items can be found in Table 1.

**TABLE 1:** Sample items from the implicit aggressiveness conditional reasoning test for college students.

Items	If, after you ask a question, the teacher does not respond immediately, you would:
Options:	(a) Suspect that the teacher is deliberately ignoring your question (aggressive).
	(b) Assume that the teacher might not have heard or is thinking about the answer (prosocial).
	(c) The teacher might not have had breakfast in the morning (illogical).
	(d) Decide to never speak again in the future (illogical).

Pre-testing the Scale: Before conducting the formal reliability and validity testing, a pre-test was carried out with a group of 30 college students to evaluate the clarity and smoothness of the scale items. During the pre-test, participants were asked to identify any ambiguous or unclear statements. Based on their feedback, several items were revised to ensure that the wording was clear and that the items effectively measured the intended constructs. This process helped improve the comprehensibility of the scale before proceeding with the formal testing phases.

#### Testing procedure and data analysis

To maintain consistency in instructions and format, we utilized the Wenjuanxing online platform for distributing the questionnaires, requiring participants to complete them using their mobile phones. We conducted descriptive statistics, correlation analysis, and exploratory factor analysis using SPSS 25.0, and performed confirmatory factor analysis using Amos 24.0.

## Results

### Content validity analysis

Drawing on the justification mechanism theory and the aggression conditional reasoning test in a professional setting, the research team identified the core elements of implicit aggression conditional reasoning through expert panel discussions. Based on this, the team systematically constructed the dimensions of the scale, including hostile attribution bias, goal derogation bias, efficacy bias, retaliation bias, oppression bias, and social discounting bias, designing corresponding items for each dimension.

Following the completion of the initial version of the scale, a panel of nine professionals, including psychology professors, doctoral, and master’s students, was invited to review the dimensions and items of the scale. They conducted multiple rounds of detailed discussions and evaluations to ensure that the items comprehensively covered the conceptual scope of implicit aggression in college students, while maintaining construct validity, clarity, conciseness, and comprehensiveness.

To validate the content validity of the scale, the panel calculated the content validity ratio (CVR) for several items in the initial scale. The results indicated that each item had a CVR value of 1, confirming the high content validity of the scale.

### Exploratory factor analysis

The study initially conducted an exploratory factor analysis (EFA) on Sample 1, employing principal component analysis and Promax oblique rotation to clearly identify the underlying factor structure. The analysis yielded a KMO value of 0.90 and a Bartlett’s Test of Sphericity result of χ^2^ = 2,455.18 (*p* < 0.001). Detailed examination of the data and the scree plot led to the extraction of six primary factors: hostile attribution bias, goal derogation bias, efficacy bias, retaliation bias, oppression bias, and social discounting bias. Loadings analysis is detailed in Table 2.

**TABLE 2:** Factor load table for exploratory factor analysis.

	Hostile attribution bias	Goal derogation bias	Efficacy bias	Retaliation bias	Oppression bias	Social discounting bias
Q1	0.76					
Q2	0.75					
Q3	0.72					
Q4		0.76				
Q5		0.69				
Q6		0.72				
Q7			0.71			
Q8			0.81			
Q9			0.78			
Q10				0.77		
Q11				0.78		
Q12				0.71		
Q13					0.77	
Q14					0.75	
Q15					0.83	
Q16						0.81
Q17						0.78
Q18						0.69

### Item analysis

An item analysis was conducted to evaluate the difficulty and discrimination of each item. The difficulty index for each item ranged from 0.3 to 0.7, indicating that the items were of moderate difficulty. Discrimination was assessed by calculating item-total correlations, with all items showing adequate discrimination (all > 0.30). Items with lower discrimination were revised or removed to improve the overall quality of the scale. These revisions were aimed at enhancing the ability of the scale to differentiate between individuals with different levels of implicit aggression.

### Confirmatory factor analysis

**FIGURE 1:**
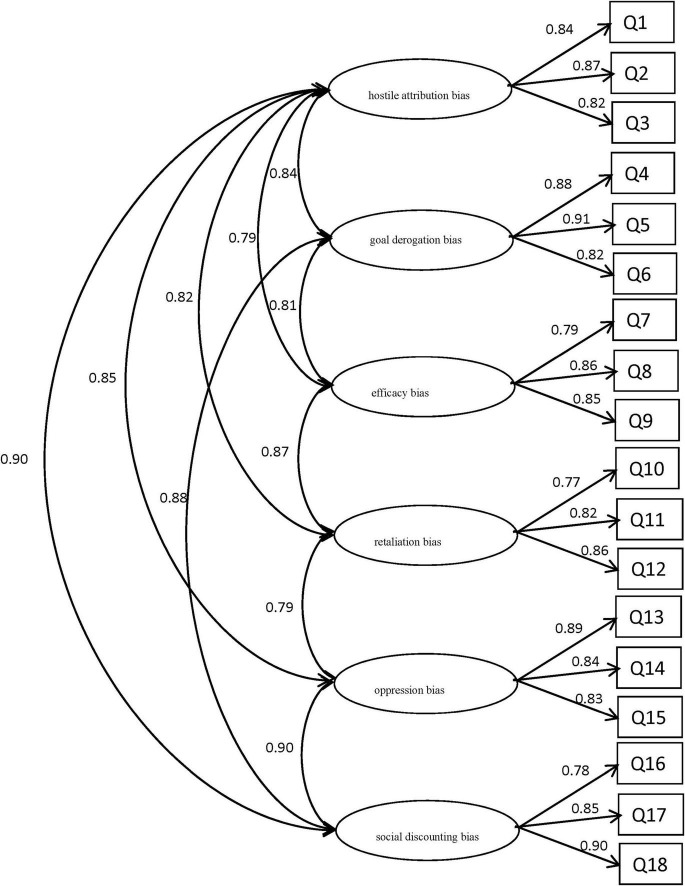
Results of confirmatory factor analysis.

To further validate the findings and the structural stability of the scale, a confirmatory factor analysis (CFA) was conducted on the results of the formal testing sample using AMOS 24.0 software for model testing and parameter estimation. The CFA results indicated good model fit, with the following fit indices meeting statistical requirements: χ^2^ = 333.82, df = 132, RMSEA = 0.08, SRMR = 0.045, NFI = 0.91, CFI = 0.92, GFI = 0.90, and PGFI = 0.66. According to commonly accepted thresholds for model fit indices—RMSEA ≤ 0.08 indicating acceptable fit, SRMR ≤ 0.05 indicating excellent fit, and NFI/CFI/GFI ≥ 0.90 indicating good fit—these values collectively support the conclusion that the model exhibits adequate-to-excellent fit. Furthermore, the PGFI value of 0.66, exceeding the commonly recommended threshold of 0.50, highlights the model’s balance between fit and parsimony. Collectively, these results provide strong evidence for the conditional reasoning test for implicit aggression in college students demonstrating both good structural validity and a well-exhausted model (Figure 1).

### Criterion-related validity

The Chinese version of the Buss-Perry Aggression Questionnaire was used as a criterion tool (Gallagher and Ashford, 2016; Li et al., 2011). In Sample 2, a Pearson correlation analysis was conducted between the total scores of the conditional reasoning test for implicit aggression in college students and the Buss-Perry Aggression Questionnaire. The analysis revealed a significant positive correlation (*r* = 0.52, *p* < 0.01), indicating that higher scores on the conditional reasoning test were associated with higher scores on the Buss-Perry Aggression Questionnaire. Specifically, the correlation coefficients between the scores of each dimension (hostile attribution bias, goal derogation bias, efficacy bias, retaliation bias, oppression bias, and social discounting bias) and the total score of the Adult Aggression Evaluation Scale ranged from 0.28 to 0.52, all reaching significant levels (*p* < 0.01).

Furthermore, correlations between the total score of the conditional reasoning test for implicit aggression and the physical aggression subscale (*r* = 0.48, *p* < 0.01), as well as the verbal aggression subscale (*r* = 0.45, *p* < 0.01) of the Buss-Perry Aggression Questionnaire, were also significant. These results further support the validity of the conditional reasoning test as an effective tool for assessing implicit aggression in college students.

In this study the reliability of the Chinese version of the BPAQ was confirmed with a Cronbach’s alpha of 0.91 for the total scale, indicating good internal consistency. The construct validity of the Chinese version of the BPAQ was further supported by a confirmatory factor analysis (CFA), which showed a good model fit with the following fit indices: χ^2^ = 345.12, df = 130, RMSEA = 0.09, NFI = 0.90, CFI = 0.91, GFI = 0.89, PGFI = 0.67. These results suggest that the Chinese version of the BPAQ maintains strong construct validity and is an effective tool for assessing aggression in college students.

### Reliability assessment

In assessing the reliability of the conditional reasoning test for implicit aggression in college students, this study employed internal consistency coefficients and split-half reliability on the formal sample data, and conducted test-retest reliability analysis on Sample 3.

Internal consistency coefficient: The internal consistency of the total scale and its six dimensions was evaluated. The Cronbach’s α coefficients for these dimensions ranged from 0.87 to 0.92, with the total scale’s internal consistency coefficient being 0.90.

Split-half reliability: The consistency of scores between the two halves of the scale was calculated as another measure of reliability. In the conditional reasoning test, the split-half reliability for each dimension remained above 0.80, and the total scale’s split-half reliability was 0.83. The high values of split-half reliability indicate good consistency between the two halves of the scale in measuring the same concept, further confirming the reliability of the scale.

Test-retest reliability: The retest data collected two weeks later showed that the correlation coefficients between the scores of the conditional reasoning test and each dimension ranged from 0.84 to 0.90, with the total scale’s test-retest reliability being 0.87. The test-retest reliability indicates that the reliability of the scale is maintained over time within a controllable range.

## Discussion

Previous measurements of college students’ aggressive behavior predominantly utilized self-report scales to assess overt aggression. However, as highlighted in the introduction, research has consistently shown that self-report scales are limited in capturing implicit aggression due to the influence of social desirability and conscious self-monitoring (James et al., 2022). These limitations underscore the need for a more objective method to assess implicit aggression, which this study sought to address through the development and validation of the conditional reasoning test for implicit aggression in college students. By providing an indirect measurement approach, this test aims to uncover the reasoning biases associated with implicit aggression, thereby offering a more reliable and nuanced tool for psychological research and clinical assessment.

The justification mechanism theory served as the theoretical foundation for constructing the conditional reasoning test, with reference to the established methodology for developing aggression-related conditional reasoning tests in professional settings. Through expert evaluation and rigorous statistical analysis, the finalized test includes six dimensions: hostile attribution bias, goal derogation bias, efficacy bias, retaliation bias, oppression bias, and social discounting bias. These dimensions were designed to capture the specific cognitive distortions that characterize implicit aggression, revealing the default response patterns of college students when faced with challenges or perceived threats. This multidimensional structure reflects a comprehensive effort to operationalize implicit aggression in a manner that is both theoretically grounded and empirically robust.

The conditional reasoning test demonstrated strong psychometric properties in terms of reliability and validity, further supporting its effectiveness as a measurement tool. The internal consistency coefficient, split-half reliability, and test-retest reliability all yielded high values, indicating that the scale is both internally coherent and stable across time. These findings align with prior research on conditional reasoning measures, such as those conducted by Galić et al. (2014) and Lee (2017), which similarly reported high reliability indices in different populations. The high reliability observed in this study reinforces the consistency and dependability of the test in assessing the dimensions of implicit aggression.

In addition to reliability, the test demonstrated strong validity, particularly in terms of content validity and convergent validity. The content validity, as assessed by the expert panel’s content validity ratio (CVR), confirmed that the items adequately represent the theoretical constructs of implicit aggression. Furthermore, the significant positive correlations between this test and the Buss-Perry Aggression Questionnaire (BPAQ) provide evidence of its convergent validity. These correlations indicate that the conditional reasoning test is not only capable of capturing implicit aggression but also reflects psychological constructs related to overt aggression, as measured by an established self-report tool. This finding bridges the gap between implicit and explicit measures of aggression, addressing a key limitation of self-report scales noted in the introduction.

Compared to previous studies, this research offers several advancements. While prior studies on conditional reasoning tests have focused primarily on internal structure and predictive validity, this study uniquely incorporated a criterion-related measure—namely the BPAQ—to evaluate convergent validity. This approach strengthens the evidence for the conditional reasoning test as a comprehensive tool for assessing implicit aggression, particularly in college students. Moreover, the incorporation of six distinct dimensions tailored to the cognitive patterns of this population enhances the specificity of the test, addressing a gap in prior research that largely centered on workplace aggression or general populations.

The findings of this study have important theoretical and practical implications. The development of the conditional reasoning test responds to the need for an objective and indirect method to assess implicit aggression, as outlined in the introduction. Since implicit aggression is not easily captured through self-report measures, this test provides a valuable alternative that can help psychologists and educators identify aggression-related cognitive patterns that may not be immediately apparent. Early identification of such patterns can inform targeted interventions, potentially reducing the risk of aggressive behaviors and improving psychological wellbeing among college students.

Future research should further validate the conditional reasoning test by examining its predictive validity in real-world contexts, such as its ability to predict aggressive behaviors or conflicts within academic and social environments. Additionally, cross-cultural studies are needed to evaluate the applicability of the test in diverse populations and cultural settings. Since aggression may manifest differently across cultural contexts, exploring these variations will enhance the generalizability and utility of the test. As noted in the introduction, the importance of understanding implicit aggression extends beyond individual assessment to broader societal implications, such as promoting safer and more inclusive environments in educational institutions.

In conclusion, this study successfully developed and validated the conditional reasoning test for implicit aggression in college students, addressing the limitations of self-report scales and contributing a novel tool to the field of psychological assessment. By combining strong psychometric properties with theoretical and practical relevance, this test provides a new avenue for understanding and addressing implicit aggression in college students. The findings underscore the value of integrating indirect measures into aggression research and highlight opportunities for future exploration to further refine and expand the test’s applications.

## Data Availability

The raw data supporting the conclusions of this article will be made available by the authors, without undue reservation.

## References

[B1] Anderson C. A. Bushman B. J. (2002). Human aggression. *Annu. Rev. Psychol.* 53 27–51. 10.1146/annurev.psych.53.100901.135231 11752478

[B2] Barksdale C. D. (2007). *Justification Mechanisms in the Conditional Reasoning Test for Aggression and Their Relation to Defense Mechanisms.* PhD diss. Knoxville, TN: University of Tennessee.

[B3] Baron R. A. Richardson D. R. (1994). *Human Aggression*, 2nd Edn. New York, NY: Plenum Press.

[B4] Bowler J. L. Bowler M. C. Cope J. G. (2013). Measurement issues associated with conditional reasoning tests: An examination of faking. *Pers. Individ. Diff.* 55 459–464. 10.1037/0021-9010.92.1.1 17227147

[B5] Buss A. H. Durkee A. (1957). An inventory for assessing different kinds of hostility. *J. Consult. Psychol.* 21 343–349. 10.1037/h0046900 13463189

[B6] Buss A. H. Perry M. (1992). The aggression questionnaire. *J. Pers. Soc. Psychol.* 63 452–459. 10.1037//0022-3514.63.3.452 1403624

[B7] Caibin D. Dequn S. Dongwei J. Lihua Z. (2023). Aggressive behavior of different types of college students with high selfesteem. *Chin. J. Health Psychol.* 31, 1102–1108. 10.13342/j.cnki.cjhp.2023.07.027

[B8] Chu K. Zhu F. (2022). Impact of effort–reward imbalance on undergraduates’ aggressive behavior: The mediating role of perceived justice and hostile attribution. *Soc. Behav. Pers. Int. J.* 50 1–10. 10.2224/sbp.11414

[B9] Didden R. Lindsay W. R. Lang R. Sigafoos J. Deb S. Wiersma J. (2016). “Aggressive behavior,” in *Handbook of Evidence-Based Practices in Intellectual and Developmental Disabilities*, ed. Singh M. N. (Berlin: Springer), 727–750.

[B10] Galić Z. (2016). Conditional reasoning test for aggression: Further evidence about incremental validity. *Int. J. Selection Assess.* 24 24–33. 10.1111/ijsa.12126

[B11] Galić Z. Scherer K. T. LeBreton J. M. (2014). Examining the measurement equivalence of the conditional reasoning test for aggression across US and Croatian samples. *Psychol. Test a Assess. Model.* 56 195–216.

[B12] Gallagher J. M. Ashford J. B. (2016). Buss–Perry aggression questionnaire: Testing alternative measurement models with assaultive misdemeanor offenders. *Criminal Justice Behav.* 43 1639–1652. 10.1177/0093854816643986

[B13] James L. R. LeBreton J. M. (2010). Assessing aggression using conditional reasoning. *Curr. Dir. Psychol. Sci.* 19 30–35. 10.1177/0963721409359279

[B14] James L. R. McIntyre M. D. Glisson C. A. Bowler J. L. Mitchell T. R. (2022). “The conditional reasoning measurement system for aggression: An overview,” in *Personality and the Prediction of Job Performance*, ed. Borman W. C. (Milton Park: Taylor & Francis Group), 271–295.

[B15] James L. R. McIntyre M. D. Glisson C. A. Green P. D. Patton T. W. LeBreton J. M. (2005). A conditional reasoning measure for aggression. *Organ. Res. Methods* 8 69–99. 10.1177/1094428104272182

[B16] Krahé B. (2020). *The Social Psychology of Aggression.* Milton Park: Taylor & Francis Group.

[B17] LeBreton J. M. Reichin S. L. te Nijenhuis J. Cremers M. van der Heijden-Lek K. (2022). Validity evidence and measurement equivalence for the Dutch translation of the conditional reasoning test for aggression. *Appl. Psychol.* 71 710–739. 10.1111/apps.12309

[B18] Lee H. J. (2017). Exploring equivalence of a Korean version of the conditional reasoning test for aggression. *Int. J. Psychol. Behav. Anal.* 2:133. 10.15344/2455-3867/2017/133

[B19] Li X. Fei L. Zhang Y. Niu Y. Tong Y. Yang S. (2011). Revision and reliability of the Chinese version of the Buss and Perry attack questionnaire. *Chin. J. Neuropsychiatric Disord.* 10 607–613.

[B20] Puhalla A. A. McCloskey M. S. (2020). The relationship between physiological reactivity to provocation and emotion dysregulation with proactive and reactive aggression. *Biol. Psychol.* 155:107931. 10.1016/j.biopsycho.2020.107931 32687869

[B21] Raine A. Dodge K. Loeber R. Gatzke-Kopp L. Lynam D. Reynolds C. (2006). The reactive–proactive aggression questionnaire: Differential correlates of reactive and proactive aggression in adolescent boys. *Aggress. Behav.* 32 159–171. 10.1002/ab.20115 20798781 PMC2927832

[B22] Thomas R. (2019). College student peer aggression: A review with applications for colleges and universities. *Aggr. Violent Behav.* 48 218–229. 10.1016/j.avb.2019.08.013

[B23] Vassar M. Hale W. (2009). Reliability reporting across studies using the Buss Durkee hostility inventory. *J. Interpers. Violence* 24 20–37. 10.1177/0886260508314931 18378813

[B24] Weltens I. Bak M. Verhagen S. Vandenberk E. Domen P. van Amelsvoort T. (2021). Aggression on the psychiatric ward: Prevalence and risk factors. A systematic review of the literature. *PLoS One* 16:e0258346. 10.1371/journal.pone.0258346 34624057 PMC8500453

[B25] Xu J. Ren J. Xiang Q. Yin X. Zhu H. Zhu F. (2023). A cross-sectional study of physical exercise and aggressive behavior in college students: The mediating role of self-efficacy and self-control. *Sichuan Sports Sci.* 5 52–58. 10.13932/j.cnki.sctykx.2023

[B26] Ye B. Ma T. Chen C. Liu M. Wang X. Yang Q. (2022). Exploring the profiles of aggressive behavior among college students: A person-centered approach. *Curr. Psychol.* 10.1007/s12144-020-01267-1

[B27] Yu H. Ma Q. Sun Y. Jiang S. Hu S. Wang X. (2024). Analyzing the effects of physical activity levels on aggressive behavior in college students using a chain-mediated model. *Cientific Rep.* 14:5795. 10.1038/s41598-024-55534-3 38461174 PMC10924895

[B28] Zeferino G. Santos, da Silva A. C. Mansur-Alves M. (2024). Instruments for evaluation of aggressiveness in sports fans: A systematic review. *Ciencias Psicol.* 18 1–16. 10.22235/cp.v18i1.3178

